# Functional Relationships between Long Non-Coding RNAs and Estrogen Receptor Alpha: A New Frontier in Hormone-Responsive Breast Cancer Management

**DOI:** 10.3390/ijms24021145

**Published:** 2023-01-06

**Authors:** Viola Melone, Annamaria Salvati, Noemi Brusco, Elena Alexandrova, Ylenia D’Agostino, Domenico Palumbo, Luigi Palo, Ilaria Terenzi, Giovanni Nassa, Francesca Rizzo, Giorgio Giurato, Alessandro Weisz, Roberta Tarallo

**Affiliations:** 1 Laboratory of Molecular Medicine and Genomics, Department of Medicine, Surgery and Dentistry ‘Scuola Medica Salernitana’, University of Salerno, 84081 Baronissi, Italy; 2 Medical Genomics Program and Division of Oncology, AOU ‘S. Giovanni di Dio e Ruggi d’Aragona’, University of Salerno, Rete Oncologica Campana, 84131 Salerno, Italy; 3 CRGS-Genome Research Center for Health, University of Salerno Campus of Medicine, 84081 Baronissi, Italy

**Keywords:** lncRNAs, estrogen receptor alpha, estrogen signaling, breast cancer biomarkers

## Abstract

In the complex and articulated machinery of the human genome, less than 2% of the transcriptome encodes for proteins, while at least 75% is actively transcribed into non-coding RNAs (ncRNAs). Among the non-coding transcripts, those ≥200 nucleotides long (lncRNAs) are receiving growing attention for their involvement in human diseases, particularly cancer. Genomic studies have revealed the multiplicity of processes, including neoplastic transformation and tumor progression, in which lncRNAs are involved by regulating gene expression at epigenetic, transcriptional, and post-transcriptional levels by mechanism(s) that still need to be clarified. In breast cancer, several lncRNAs were identified and demonstrated to have either oncogenic or tumor-suppressive roles. The functional understanding of the mechanisms of lncRNA action in this disease could represent a potential for translational applications, as these molecules may serve as novel biomarkers of clinical use and potential therapeutic targets. This review highlights the relationship between lncRNAs and the principal hallmark of the luminal breast cancer phenotype, estrogen receptor α (ERα), providing an overview of new potential ways to inhibit estrogenic signaling via this nuclear receptor toward escaping resistance to endocrine therapy.

## 1. Introduction

Breast cancer (BC) is the most diagnosed cancer among women and is a leading cause of death [1]. BC heterogeneity is strictly correlated with its multiple subtypes, which originate from different cells with a proper function. Tumor-forming cells derive mainly from the epithelium of the ducts or lobules that, following genetic and epigenetic alterations, undergo uncontrolled growth and the dysregulation of intracellular signaling [2,3]. The majority of BCs are considered hormone-responsive based on the expression of hormone receptors, among which the alpha receptor subtype is clinically relevant for endocrine therapy. Indeed, estrogen receptor α (ERα) has been for a long time the main therapeutic target due to its key role in the regulation of genes involved in cellular processes such as proliferation, apoptosis, invasion, metastasis, and angiogenesis [4,5]. The primary goal of endocrine therapy for ERα positive (ERα+) patient management is the blocking of estrogen signaling through the inhibition of estrogen biosynthesis by aromatase inhibitors (AIs) or by using antiestrogen drugs. The latter are grouped into two main classes: selective estrogen receptor modulators (SERMs), such as tamoxifen or raloxifene, and selective estrogen receptor down-regulators (SERDs), such as fulvestrant (ICI 182,780) [6,7]. Although these therapies show effectiveness in clinical practice, de novo or acquired resistance to hormone therapy remains the main problem associated with disease recurrence and progression. Several studies have explored different resistance mechanisms leading to the estrogen-independent growth of hormone receptor-positive BC following genetic and epigenetic alterations. These mechanisms include, for example, acquired mutations of the ESR1 gene in response to endocrine loss, the constitutive activation of cyclin-dependent kinases (CDK4-6), the dysregulation in the cross-talk between ERα and growth factor receptor signaling, epigenetic alterations and modifications by histone deacetylase (HDAC), as well as interactions with the tumor microenvironment and host immune response [8]. Among these, alterations in long non-coding RNA (lncRNA) expression patterns were identified as involved in different mechanisms, such as drug resistance, occurrence, and progression in multiple cancer histotypes, including BC [9]. LncRNAs belong to the large class of non-coding transcripts and, recently, have drawn attention for their functional roles in epigenetics and transcriptional and post-transcriptional processes [10], rendering them very attractive for the improvement of cancer treatment efficiency. The heterogeneity of lncRNA mechanisms of action depends on multiple factors, such as their cellular localization and interacting molecules [11].

In this review, we described the functional relationship between ERα and lncRNAs, highlighting their contribution to the regulation of crucial BC cell signaling activities. Interestingly, in this context, the binding of ERα by estrogens or the absence of hormone stimulation showed different contributions to BC progression and cancer cell survival, suggesting a new landscape that might be studied and exploited for novel potential therapeutic target identification.

## 2. Ligand-Induced and Constitutive Activity of ERα

As a member of the nuclear hormone receptor superfamily, ERα is engaged in a plethora of mechanisms that could act in parallel or be altered in a tumor context, thus providing several ways to escape from the attempts to block its mitogenic activity. Furthermore, the ability of estrogen receptors to act even in the absence of hormone stimulation leads to increased challenges to functional mechanism understanding.

The knowledge of how Erα’s protein structure relates to its activity and how this affects BC cell biological functions is critical for new drug development aiming at preventing or overcoming endocrine resistance. ERα is composed of six functional domains (A-F), including the hormone binding domain (HBD) located within the E region at the C-terminus. The well-described estrogen-dependent pathway foresees that the binding with specific ligands allows ERα to migrate into the nucleus where, acting as a transcription factor, it modulates target gene expression through either direct or indirect binding to specific chromatin loci [12].

Several lines of evidence showed that, in the absence of hormones, a plethora of post-translational modifications (PTMs) or extracellular signals are able to mediate ERα activity, comprising growth factors, cytokines, and other hormones [13] able to maintain the basal transcriptional activity of the receptor. The unliganded receptors also bind to multiple chromatin sites, thus ensuring the constitutive expression of several coding and non-coding RNAs [14,15] to promote cell development and epithelial phenotype maintenance. Among the ERα estrogen-independent functions, RNA processing activity was observed [16]; this activity was also shared by ligand-activated receptors [17], suggesting a new landscape that might be studied and exploited for novel potential therapeutic target identification.

Both in ligand-induced and hormone-independent receptor activity, ERα plays a central role in breast carcinogenesis by interacting with a multitude of co-regulators to modulate target gene transcription. Once in the nucleus, ERα becomes a key component of multiple protein complexes involved in crucial functions for tumor cell proliferation and survival. Interestingly, a substantial fraction of ERα molecular partners is essential for cancer cell survival [18], as they are involved in transcriptional machinery assembly, splicing events and epigenetic modifications [19]. Most ERα-associated co-regulators were shown as ligand-specific, and the functional interplay among the molecules is often mediated by RNA-protein interactions [20,21]. Among the RNA molecules, the class of non-coding RNAs is increasingly attractive for novel approaches in the treatment of cancer, as their deregulation was specifically linked to known tumors due to their tissue specificity, as well as to their association with cancer hallmarks [22,23].

## 3. lncRNAs and Cancer

Cancer is mostly due to genetic mutations affecting gene networks to different extents [24]. Many of these mutations lie within the non-coding regions of the genome that carry out their functions as RNA molecules [25]. Indeed, despite the fact that ~70% of the human genome is transcribed, only 2% comprises protein-coding gene sequences [26]. The remaining non-coding transcripts are classified according to their length; most of them are small molecules (small non-coding RNAs, sncRNAs), but others may be more than 200 nucleotides in length and, for this reason, they are called long non-coding RNAs (lncRNAs) [27]. lncRNAs are receiving growing concerns in human diseases, particularly cancer. Next-generation sequencing (NGS) has revealed thousands of lncRNAs whose aberrant expression is associated with different cancer types, including BC [27].

They are generally defined as molecules longer than 200 nucleotides with many features shared with mRNAs since they are transcribed by RNA polymerase II, 5′ capped, polyadenylated, spliced, and, in many cases, exported from the nucleus, but they lack open reading frames (ORFs) [28,29]. Currently, there are different classification methods that group lncRNAs depending on major features, which are based on their genomic location, effects exerted on DNA sequences, and mechanisms of action. According to their position within the genome, lncRNAs are classified into five categories, namely, sense, antisense, bi-directional, intronic, and intergenic lncRNAs [30].

Recent findings have revealed that lncRNAs are implicated, at different stages, in cancer development by interacting with DNA, RNA, proteins, or a combination of these. The mechanisms by which lncRNAs contribute to the modulation of regulatory pathways that promote cancer development are different. Indeed, an increasing number of studies reported that lncRNAs play a pivotal role in regulating all steps of gene expression, such as transcription, post-transcription, translation, and epigenetic modification [31]. In the latter case, it is believed that lncRNAs exert, above all, a repressive action against target genes through histone modifications, the remodeling of chromatin structures, or DNA methylation. For instance, the most well-characterized lncRNAs, such as HOTAIR, linc-ROR, ANRIL, H19, and XIST, repress gene transcription by recruiting histone-modifying or chromatin-remodeling proteins [32]. Additionally, lncRNAs could act as oncogenes or tumor-suppressing factors, adding a new layer of complexity to the molecular architecture of human cancers [33].

## 4. Current Methods for the Functional Analysis of lncRNAs 

The elucidation of the molecular mechanisms underlying the action of lncRNAs represents a challenging task that may be accomplished by the application of multiple assays, briefly summarized below (Table 1). The determination of the intracellular localization of lncRNAs represents an approach providing the first hint of possible lncRNA-specific cellular functions and may be fulfilled by the application of methods such as quantitative PCR (qPCR) [34], RNA-fluorescent in situ hybridization (RNA-FISH) [35], RNA-FISH combined with stochastic optical reconstruction microscopy (STORM) [36], and lncRNA labeling with aptamers linked to fluorescent tags [37,38]. A more profound functional characterization may be achieved after lncRNA knock-down mediated by RNA silencing with small interfering RNAs (siRNAs), short hairpin RNAs (shRNAs), antisense oligonucleotides (ASOs), or via knock-outs/knock-ins using CRISPR/Cas9 technologies [39]. Another important milestone in the discovery of lncRNA functions is the establishment of secondary and three-dimensional lncRNA structures. Chemical or enzymatic probing, shotgun secondary structure fragment analysis [40], or computational prediction [41,42] are the most popular approaches adopted to uncover secondary structures. The determination of three-dimensional structures and tertiary lncRNA interactions, instead, is generally achieved using solution-state nuclear magnetic resonance (NMR) spectroscopy [43,44], small-angle scattering (SAS) [45], X-ray diffraction, or cryo-electron microscopy (reviewed in [39]). Finally, computational modeling may be applied to ascertain insights into secondary and 3D RNA structure prediction [46,47]. Since lncRNAs do not act alone but in association with proteins or within multi-molecule complexes, the determination of lncRNA-protein interactions (LPIs) is crucial to understanding their molecular functions. Based on whether the protein or RNA component of the lncRNA-protein complex is targeted, protein-centric and RNA-centric LPI determination assays are distinguished (see [48] for a detailed overview).

Protein-centric assays take advantage of immunoprecipitation followed by RNA extraction that may be further analyzed using quantitative PCR (qPCR), microarrays, or high-throughput sequencing. Many of these methods are based on the cross-linking immunoprecipitation (CLIP) assay [49,50], which provides evidence of lncRNA-protein interactions. In case the location of a protein binding site/sites within a lncRNA and the relative differences in their binding strengths are needed, methods, such as TRIBE (targets of RNA-binding proteins identified by editing) [51] and DO-RIP-seq (digestion-optimized RNA immunoprecipitation cDNA library sequencing) [52] may be applied.

Concerning RNA-centric methods, the determination of proteins bound to an RNA of interest may be fulfilled in vitro with RNA-affinity purification followed by mass spectrometry [53,54,55,56], protein microarrays [57], or in vivo using in-cell RNA-protein cross-linking followed by the isolation of target lncRNA-protein complexes with biotinylated antisense probes [54,58,59] or peptide nucleic acid oligomers [55]. Otherwise, a histidine-biotin (HB) tag may be attached in vivo to the lncRNA of interest, thus allowing efficient HB-tag-based affinity RNA purification [56]. In case the experimental plan aims to interrogate lncRNA-chromatin interactions, chromatin isolation with RNA purification (ChIRP) [59,60] or RNA chromosome conformation capture (R3C) [61] may be applied to map lncRNA binding sites within the genome.

In the end, the biophysical characterization of quantitative and qualitative LPIs may be achieved via the application of methods such as the electrophoretic mobility shift assay (EMSA) [62], filter-binding assays [62], or surface plasmon resonance [63,64].

The data obtained downstream of the applications of LPI determination methods are collected in databases of lncRNA-protein interactions (Starbase [65], RNAInter [66], POSTAR [67], NPInter [68], and RAIN [69]) or RNA-binding motifs (ATtRACT [70] and oRNAment [71]). These curated databases are further exploited by LPI prediction algorithms that use molecular docking or machine learning approaches that were extensively described by Philip et al. [72].

Intriguingly, recent studies have indicated that some lncRNAs contain small open reading frames (less than 300 nucleotides long) encoding for short peptides that may regulate key biological processes, such as muscle function, mRNA stability, and gene expression (reviewed in [73,74]). Since these lncRNAs combine both protein-coding and non-coding functions, they were suggested to be reclassified as bi-functional RNAs [73]. Several studies demonstrated the importance of this lncRNA subtype in TNBC development. In particular, it was shown that translated from LINC00665, micro peptide CIP2A-BP inhibits the metastatic potential of cancer cells [75], whereas lncRNA LINC00908 encodes a 60-aa polypeptide ASRPS inhibiting angiogenesis in mouse xenografts and spontaneous BC models [76]. Methods for the identification of lncRNA-encoding peptides are based on ribosome profiling, mass spectrometry, and global translation initiation sequencing (GTI-seq) mass spectrometry (reviewed in [77]). Moreover, several computational methods for the prediction of long non-coding RNA peptides were developed (reviewed in [74]).

**Table 1:** Selected methods for functional lncRNA analysis.

Method	Reference
**Determination of the intracellular lncRNA localization**	
Quantitative PCR (qPCR)	[34]
RNA-fluorescent in situ hybridization (RNA-FISH)	[35]
RNA-FISH combined with stochastic optical reconstruction microscopy (STORM)	[36]
lncRNA labeling with aptamers linked to fluorescent tags	[37,38]
**lncRNA depletion or over-expression**	
Small interfering RNA (siRNA) silencing	[78,79]
Short hairpin RNA (shRNA) silencing	[80]
Antisense oligonucleotide (ASO) silencing	[79]
CRISPR/Cas9 knock-out/knock-in	[81]
**The establishment of secondary and three-dimensional lncRNA structures**	
Shotgun secondary structure fragment analysis	[40]
Solution-state nuclear magnetic resonance (NMR) spectroscopy	[43,44]
Small-angle scattering (SAS)	[45]
X-ray diffraction and cryo-electron microscopy	[39]
Computational prediction	[41,42,46]
**The determination of lncRNA-protein interactions (LPIs)**	
Cross-linking immunoprecipitation (CLIP)	[49,50]
Targets of RNA-binding proteins identified by editing (TRIBE)	[51]
Digestion-optimized RNA immunoprecipitation cDNA library sequencing (DO-RIP-seq)	[52]
RNA-affinity purification followed by mass spectrometry	[53,54,55,56]
RNA-affinity purification followed by protein microarrays	[57]
The isolation of target RNA molecules by biotinylated antisense probes	[54,58]
The isolation of target RNA molecules by peptide nucleic acid oligomers	[55]
HB-tag-based affinity RNA purification	[56]
Chromatin isolation with RNA purification (ChIRP)	[59,60]
RNA chromosome conformation capture (R3C)	[61]
LPI computational prediction	[72]
**The biophysical characterization of quantitative and qualitative LPIs**	
Electrophoretic mobility shift assay (EMSA)	[62]
Filter-binding assays	[82]
Surface plasmon resonance	[63,64]
**The evaluation of the coding capacity of lncRNAs**	
Ribosome profiling	[83]
Mass spectrometry	[84]
Global translation initiation sequencing (GTI-seq)	[73]

## 5. LncRNA Mechanisms in Breast Cancer 

Since these molecules appear to be involved in multiple biological processes, such as cell proliferation, differentiation, chromosome remodeling, epigenetic modulation, and transcriptional and post-transcriptional modifications [85,86], understanding their regulation would provide new insights into cancer biology. Recent studies have proved that some lncRNAs are abnormally up-regulated in a variety of BC cell lines, and their altered expression may contribute to cancer initiation and progression [87]. Other evidence also demonstrates that estrogen stimulation affects the expression levels of many lncRNAs in BC [88]. Specifically, lncRNAs could act as either promoters or inhibitors of metastasis, but the complete scenario by which they concur to BC initiation is still far from being completely unraveled. In hormone-responsive BC, understanding the functional cooperation between lncRNAs and ERα could be useful for the identification of specific mechanisms involved in antiestrogen resistance, thus providing alternative ways to escape it. Moreover, since one of the most adopted endocrine treatments is represented by aromatase inhibitors, which deplete the organism of estrogens, it is also important to understand the functional link between breast-acting lncRNAs and unliganded (constitutive) ERα.

**Figure 1:**
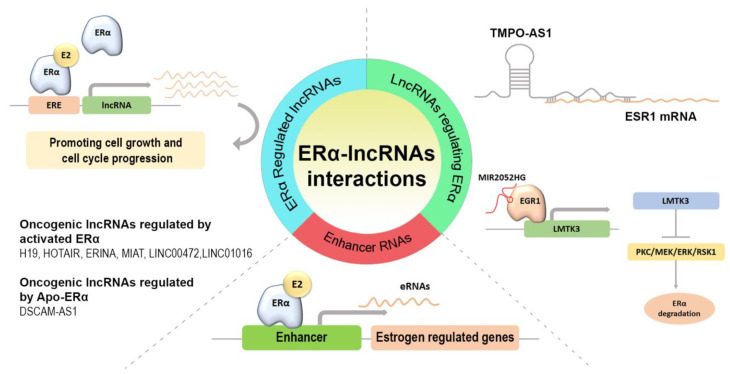
Schematic representation of the ERα-lncRNAs interactions reviewed in the text.

In Table 2 and Figure 1, all the lncRNAs whose roles were investigated in this review are summarized. 

### 5.1. Estrogen-Inducible lncRNAs

The application of NGS methods, particularly GRO and RNA-Seq, allowed the identification of a huge number of lncRNAs, many of them appearing to be regulated by estrogenic signaling or having specific roles in estrogen-dependent transcriptional regulation.

#### 5.1.1. lncRNA H19

The maternally imprinted oncofetal lncRNA H19, physiologically expressed during embryogenesis and down-regulated at birth, was demonstrated to be re-expressed in a variety of cancers and act through different mechanisms [89]. Aberrant expression levels of H19 were identified in different cancer types, including BC, where it acts as an oncogenic factor [90]. A relationship between H19 expression and hormone receptors (both PR and ER) was previously proposed [91]. H19 appeared to be over-expressed in Erα+ MCF7 cells compared to the Erα- MDA-MB-231 cell line [92]. Sun et al. demonstrated that stimulation with 17β-estradiol produced a dose and time-dependent induction of H19 expression in MCF-7 cells. This effect was supposed to be Erα-mediated since Erα inhibition, either through the treatment with ICI 182,780, the specific Erα antagonist, or knock-down, determined its reduction [92]. On the other hand, H19 knock-down decreased BC cell survival and blocked estrogen-induced cell growth, while its over-expression induced cell proliferation [92].

Another study demonstrated the involvement of H19 in the symmetric division of breast cancer stem-like cells (BrCSCs), which resulted in increasing levels of self-renewing [93]. BrCSCs are highly implicated in tumor generation, resistance, and recurrence and, in the cited study, it was demonstrated that H19 inhibited Let-7c expression by acting as a ceRNA (competing endogenous RNA), thus affecting the estrogen-activated Wnt pathway and determining the BrCSC symmetric division [93].

#### 5.1.2. HOX Transcript Antisense RNA (HOTAIR)

Similar to H19, the expression of the lncRNA HOX transcript antisense RNA (HOTAIR) was regulated in an estrogen-dependent manner [94]. HOTAIR belongs to the mammalian HOX locus, and its structure is devoid of any stem-loops, suggesting this is a pre-miRNA [95]. This lncRNA could be considered a potential diagnostic and clinically actionable marker for different cancer types [95], including BC since its expression profile appears to be up-regulated in both primary tumors and distant metastases compared to adjacent normal tissue [96]. HOTAIR is transcribed from the antisense strand of the HOXC gene locus in chromosome 12, and its promoter contains multiple functional estrogen-response elements (EREs) [94].

From a functional point of view, HOTAIR is involved in epigenetic regulation and plays an important role in several cellular pathways by interacting with polycomb repressive complex 2 (PRC2) [95], which modulates epigenetic silencing in several processes, including neoplastic transformation [97]. PRC2 is a histone methyl transferase complex mainly composed of three major subunits: EZH2, the key factor for methyl transferase activity, SUZ12 and EED, which increase EZH2 RNA binding affinity [95]. HOTAIR localizes and targets PRC2 genome-wide [98] and functions as a molecular scaffold, also interacting with the LSD1 (lysine-specific demethylase 1) complex to regulate gene expression by affecting histone H3 demethylation at lysine 4 [95]. HOTAIR binds PRC2 to the 5’ domain and LSD1 to the 3’ domain affecting, through these two complexes, chromatin remodeling and the expression of different genes involved in a variety of cell functions [98,99]. 

Concerning estrogenic signaling, Bhan et al. identified the mechanism by which Erα recruits multiple ER-coregulators, such as histone methylases MLL1 and MLL3 and CBP/p300, and binds the HOTAIR promoter region in an E2-dependent manner [94]. Particularly, the HOTAIR promoter is targeted by histone H3K4-trimethylation, histone acetylation, and RNA polymerase II in the presence of E2; on the contrary, the knock-down of both Erα and MLLs down-regulates E2-induced HOTAIR expression [94]. 

#### 5.1.3. lncRNA ERINA

The intergenic lncRNA ERINA (estrogen-inducible lncRNA) was identified to be highly expressed in multiple cancer types, especially in Erα+ BC [100]. ERINA was described as an estrogen-responsive oncogenic factor because its knock-down inhibits cell-cycle progression and cancer cell proliferation both in vitro and in xenograft in vivo models, while its over-expression promotes cell growth and cell-cycle progression [101].

This functions as an ERα-responsive gene: an ER-binding element was identified within the ERINA intronic site, and the enrichment of H3K27Ac suggested that this region functions as an enhancer to mediate the estrogen-responsive induction of ERINA [101]. 

Its oncogenic roles were found to be directly related to an interaction with E2F transcription factor 1 (E2F1). ERINA over-expression induces the sequestration of tumor suppressor retinoblastoma protein 1 (RB1), which normally binds to E2F1, and the release of E2F1, causing the over-expression of its target genes particularly involved in cell-cycle progression [101]. These findings also suggested that ERINA over-expression may contribute to drug resistance and the poor survival of patients with ERα+ BC not responding to endocrine therapies [101].

#### 5.1.4. Myocardial Infarction-Associated Transcript (MIAT)

Another lncRNA regulated via estrogenic signaling is the myocardial infarction-associated transcript (MIAT) [102]. 

Significant evidence points to the involvement of MIAT in various diseases and cellular processes [103,104,105,106], and the abnormal expression of this lncRNA was observed in multiple malignancies and in BC [107,108].

Concerning BC, its over-expression was more likely observed in ERα+ BC tissues than in ERα- ones [102]. Li et al. demonstrated that estrogen signaling activation using diethylstilbestrol (DES) allows the dose and time-dependent up-regulation of MIAT in MCF-7 cells. ERα inhibition through either silencing or pharmacological blockade with the specific antagonist ICI 182,780 proved that this occurred through an ERα-dependent mechanism. Moreover, MIAT knock-down allowed decreased DES-induced MCF-7 cell proliferation, while its over-expression increased MCF-7 cell growth [102]. In another study, it was demonstrated that MIAT knock-down allowed the suppression of the epithelial-mesenchymal transition (EMT), decreased the migration and invasion of MCF-7 BC cell lines, and inhibited tumor growth in vivo [109]. In this study, MIAT was described as a ceRNA in the modulation of the tumor suppressor dual specificity phosphatase 7 (DUSP7) by uptaking miR-155-5p [109]. 

#### 5.1.5. Long Intergenic Non-Protein Coding RNA 472 (LINC00472) and Long Intergenic Non-Protein Coding RNA 1016 (LINC01016)

Among the lncRNAs regulated by ERα, LINC00472 and LINC01016 were also identified [96]. 

The expression of intergenic LINC00472 correlates with BC progression and patient survival; particularly, its over-expression was found to be associated with low tumor grade, early-stage disease, estrogen or progesterone receptor positivity, and less cancer aggressiveness [110], while lower expression was associated with aggressive tumor features and unfavorable disease outcomes [110,111].

In addition, it was demonstrated to be estrogen-responsive, and an ERα-binding site within its promoter region was predicted and confirmed [111]. Indeed, LINC00472 is up-regulated by ERα, and its inhibition correlates to poor tumor growth and improved patient outcomes [96].

The second intergenic lncRNA, LINC01016, was identified to be highly expressed in BC and a direct ERα transcriptional target [112] since the receptor binds within its promoter region [113]. Its expression correlates with ERα expression in clinical samples and shows prognostic significance in relation to patients’ survival; its over-expression was observed more specifically in ERα+ tumors with more favorable clinical outcomes [114].

#### 5.1.6. lncRNA DSCAM-AS1 Regulation by Unliganded ERα

Among ERα activities, it is important to define its constitutive regulatory role in the absence of ligand stimulation. This receptor plays a hormone-independent function in the maintenance of BC cell’s epithelial phenotype. Miano et al. reported the lncRNA DSCAM-AS1 among the genes specifically regulated by unliganded ERα (Apo-ERα) in MCF-7 cells [113]. DSCAM-AS1 is a cancer-related lncRNA over-expressed in luminal A, B, and HER2-positive BCs [115]. This lncRNA is implicated in multiple tumorigenic processes, including DNA replication and chromosome segregation [96]. In another study, further evidence of DSCAM-AS1 expression regulated by ERα was demonstrated [116]. In particular, the authors demonstrated the interaction between ERα and the DSCAM-AS1 promoter and how the association between DSCAM-AS1 and hnRNPL led to a more aggressive cancer phenotype [116]. In addition, the knock-down of DSCAM-AS1 reduced the growth of ERα+ BC cells, diminished EMT markers, and limited cell colony formation [96,113]. 

### 5.2. lncRNAs Able to Regulate ERα Expression

lncRNAs are involved in different steps of gene expression, including transcription, mRNA stability, translation, and epigenetic modifications [117]. Among the lncRNAs that stabilize ERα mRNA, TMPO antisense RNA1 (TMPO-AS1) was identified by Mitobe et al. [118]. Its functions are widely recognized in various diseases, especially in human cancers, and previous studies demonstrated that this acts as an oncogenic factor in colorectal cancer, osteosarcoma, cervical cancer, and non-small cell lung cancer [119]. 

TMPO-AS1 stabilizes ERα transcripts and positively regulates the expression of the receptor through direct binding to ESR1 mRNA [118]. In this way, TMPO-AS1 promotes cell growth and proliferation of ERα+ BC cells both in vivo and in vitro [118]. 

Another mechanism involved in ERα stability maintenance was defined for lncRNA MIR2052HG [120], which foresees that this lncRNA regulates lemur tyrosine kinase 3 LMTK3, which is responsible for ERα stability through the PKC/MEK/ERK/RSK1 axis via EGR1 (early growth response protein 1) [120]. Functionally, MIR2052HG interacts with EGR1 and facilitates its recruitment to the LMTK3 gene promoter. On its end, LMTK3 maintains ERα levels both by reducing protein kinase C (PKC) activity, determining an increment of ESR1 transcription through AKT/FOXO3, and by reducing ERα degradation mediated by the PKC/MEK/ERK/RSK1 axis [120]. To confirm this evidence, the depletion of MIR2052HG in BC cells decreased LMTK3 expression and cell growth [120].

In summary, MIR2052HG directly interacts with the EGR1 protein, enhancing LMTK3 transcription and thus sustaining ESR1 expression and stabilizing ERα protein [120]. Furthermore, the inhibition of MIR2052HG in BC cell lines could decrease ERα expression and cell proliferation [121].

### 5.3. Enhancer RNAs (eRNAs)

In the beginning, enhancers were defined as DNA fragments located on chromatin, controlling transcription as cis-acting factors. Subsequently, the transcripts derived from active enhancers were identified, and these were named enhancer RNAs (eRNAs) [122,123,124,125]. Enhancer RNAs (eRNAs) may be lncRNAs transcribed bi-directionally by polymerase II from the DNA sequences of enhancer regions marked by H3K27ac and H3K4me1 [126]. Their function is not well defined, but it is known that these lncRNAs are able to increase the expression of target genes and stabilize the binding of active transcription factors when stimulated [126]. On the one hand, eRNAs link DNA enhancers generating them, to target gene promoters, thus aiding functional chromosome architecture formation [127]. On the other hand, they act during the release of paused RNA polymerase II in order to induce transcriptional activation [128,129]. 

In estrogenic signaling, active ERα is predominantly located at the enhancer regions [130,131]. Genome-wide studies showed that this receptor, after the activation by 17β-estradiol, could induce a global increase in the transcription of eRNAs close to the enhancers of estrogen-regulated coding genes [127]. Notably, it confirmed the existence of two categories of enhancers, both of which showed strong ERα binding and generated RNAs that could be activated or repressed following estrogen exposition [132]. Using the genome-wide nascent transcript profiles in BC cells, Yang et al. identified a group of eRNAs essential for estrogen-induced transcriptional repression [132]. In particular, they described the mechanisms by which the eRNAs TM4SF1 and EFEMP1 not only stabilize promoter-enhancer interactions but also recruit ERα to the enhancer regions to facilitate the formation of a functional transcriptional complex and promote the association of the histone demethylase KDM2A, which dismisses RNA polymerase II from the designated enhancers and suppresses the transcription of target genes [132]. ERα directly binds eRNAs through its DNA-binding domain [132].

In another study using ChIP-Seq, a global profile of ERα co-activator thymine DNA glycosylase (TDG), which plays an essential role in DNA demethylation, was generated in response to 17β-estradiol in the MCF7 BC cell line [133]. Following estrogen stimulation, TDG was co-recruited with ERα, RNA Pol II, and other co-regulators to enhancer regions marked by histone modifications indicative of active enhancers [133]. Contrarily, TDG depletion inhibited the estrogen-mediated transcription of eRNAs and the transcription of ERα-target genes [133].

**Table 2:** Cellular functions of lncRNAs in BC.

LncRNAs	Expression in BC	Regulation	Cellular Functions	References
lncRNA H19	Up-regulation	Estrogen-dependent	Proliferation, tumorigenesis, migration, invasion, and EMT	[90,92,93]
HOTAIR (HOX transcript antisense RNA	Up-regulation	Estrogen-dependent	Proliferation, invasion, migration, survival, epigenetic regulation, and chemotherapy resistance	[94,97]
LncRNA ERINA	Up-regulation	Estrogen-dependent	Proliferation, survival, and chemotherapy resistance	[101]
MIAT (myocardial infarction-associated transcript)	Up-regulation	Estrogen-dependent	Proliferation, migration, invasion, chemotherapy resistance, and EMT	[102]
LINC00472	Up-regulation	Estrogen-dependent	Proliferation, survival, migration, and invasion	[110,111]
LINC01016	Up-regulation	Estrogen-dependent	Proliferation and survival	[114]
LncRNA DSCAM-AS1	Up-regulation	Estrogen-independent	Tumorigenic processes, DNA replication, chromosome, segregation, survival, and EMT	[113,116]
TMPO-AS1 (TMPO antisense RNA1)	Up-regulation	Regulation of ERα expression	Proliferation and cell growth	[118]
MIR2052HG	Up-regulation	Regulation of ERα expression	Proliferation and cell growth	[120,121]

## 6. Prognostic and Clinical Significance of lncRNAs in Hormone-Responsive BC Treatments

The inhibition of estrogenic signaling with ET is an effective treatment for ERα+ BC tumors. Unfortunately, the development of endocrine therapy resistance (ETR) is a frequent event resulting in disease relapse and decreased overall patient survival. In the context of lncRNAs, some of these molecules were described as involved in ETR mechanisms. 

Estrogen-regulated lncRNA H19 was previously described to play a significant role in the estrogen-induced proliferation of ERα+ BC cells. In addition, its involvement in ETR mechanisms was also observed. This lncRNA seems to be important also for the proliferation and survival of ET-resistant cells [134]. The treatment of resistant cells with tamoxifen or fulvestrant allows for increments in H19 expression, while its decreased expression overcomes resistance phenomena in these cells [134]. Basak et al. demonstrated that, in ETR cells, H19 regulates ERα expression at both the mRNA and protein levels; particularly, H19 protects the receptor against fulvestrant-mediated down-regulation [134]. In these cell lines, H19 expression was found to be regulated by Notch and HGF signaling, and the pharmacological inhibition of these pathways significantly reverted the resistance to tamoxifen and fulvestrant in an H19-dependent manner [134]. In summary, H19 expression acts as an ERα modulator, and subsequently, ERα levels could be decreased by blocking Notch and c-MET receptor signaling, which helps in overcoming the resistance to fulvestrant and tamoxifen [134]. Another study demonstrated that H19 knock-out allows the down-regulation of EMT-related transcription factors in tamoxifen-resistant BC cells through the inhibition of Wnt/β-catenin pathway activation [135]. 

The lncRNA HOTAIR was described to be a predictor of adverse outcomes in cancer; in BC, its high expression was linked to the mechanisms of metastasis and drug resistance, especially in eRα+ tumors [136]. A higher expression level of this lncRNA was observed in tamoxifen-resistant cells, while its down-regulation inhibited the colony formation abilities of these cells [136].

The lncRNA urothelial carcinoma-associated protein1 (UCA1) was shown to have high expression levels in tamoxifen-resistant BC cells, where it conferred resistance by regulating the EZH2/p21 axis [137]. UCA1 expression was also detected in the exosomes released from tamoxifen-resistant BC cells; treating MCF-7 BC cells with these exosomes in vitro resulted in increased resistance to tamoxifen in this model [138]. Furthermore, UCA1 could also activate PI3K/AKT/mTOR signaling, which is known to be involved in tamoxifen-resistance acquisition [139].

Another lncRNA associated with the ETR mechanism is MAFG-AS1, whose over-expression is associated with poor prognoses in ERα+ BC. Moreover, the down-regulation of this lncRNA determines cell proliferation inhibition and apoptosis induction [140]. The gene locus of MAFG-AS1 contains an ERE element [140]. Functionally, a cross-talk between estrogenic signaling and cell-cycle regulation by MAFG-AS1 and CDK2 that could promote tamoxifen resistance was described. This mechanism promotes CDK2 expression by sponging miR-339-5p; both MAFG-AS1 and CDK2 were found to be up-regulated in tamoxifen-resistant cells [140].

In addition, lncRNAs may act as miRNA molecular sponges to promote drug resistance in BC. An example was represented by the lncRNA ROR, whose decreased expression could attenuate the resistance of BC cells to tamoxifen [141]. This lncRNA acts as a molecular sponge of miR-205 to increase the expression of ZEB1 and ZEB2, promoting EMT and tamoxifen resistance [142]. In another study, it was demonstrated that ROR could induce tamoxifen resistance by activating MAPK/ERK signaling; ROR promoted the degradation of dual specificity phosphatase7 (DUSP7), which is an important inhibitor of MAPK/ERK signaling, leading to tamoxifen resistance [143].

## 7. Conclusions

A recent study defined the novel and interesting role of ERα acting as an RNA-binding protein [17]. This finding opened a window above the other classes of Erα-interacting partners, extending its functional roles not only in the nuclear compartment but also in the cytoplasm. Xu et al. reported how ERα, by directly binding to specific mRNAs in the cytoplasm, promotes BC progression and resistance to drug therapies [17]. Among the several RNA molecules that ERα may bind to, there are also non-coding RNAs and particularly lncRNAs [17], whose implications in BC progression and induction were previously demonstrated. Recently, the ERα-lncRNA interaction was further investigated from a biochemical point of view, identifying the amino acid region of ERα as most likely involved in the direct association with RNA molecules [144]. This adds another level of complexity to the already complicated regulation of estrogen signaling by ERα. Indeed, in addition to the most investigated functional cooperation, the direct interaction between the estrogen receptor and specific lncRNA molecules could profoundly influence the transcriptional and post-transcriptional activity of the receptor, its chromatin binding, and the possible association with nascent transcripts, the stabilization of target mRNAs, their nucleus-cytoplasm translocation, and the consequent modulation of protein expression levels. Although there is still much to clarify about the functional roles of lncRNAs in BC, due to their involvement in estrogenic signaling, they offer a novel opportunity in the field of molecular targeted therapy that may escape the mechanisms of endocrine resistance. Indeed, if compared to mRNAs, lncRNAs are more stable and tissue-specific, rendering these molecules also potential biomarkers for the early diagnosis of BC and putative therapeutic targets [145]. For example, their different expression levels could help to discriminate between normal and tumor tissues or the different stages of BC [146]. Despite huge advances, there are still many challenges that must be faced and overcome; the major limiting factor concerning the application of lncRNAs in clinical practice is the lack of effective detection, off-target effects, the high number of their isoforms, the design of specific small molecule drugs, and their delivery methods [147]. Nowadays, several research lines are exploring novel valid strategies to target lncRNAs in order to improve their detection in the circulatory system [148]. The main lncRNA-based clinical approaches involve the application of RNAi-mediated gene silencing, the use of synthetic oligonucleotides, such as locked nucleic acid-modified oligonucleotides (LNAs), or the application of genome editing tools, such as the CRISPR/Cas9 system to specifically silence oncogenic lncRNAs [146]. Although there is still much to investigate, in this review, the interactions between some well-characterized lncRNAs and ERα were discussed, particularly paying attention to the molecular mechanisms by which these ncRNAs are regulated or regulate estrogenic signaling and unearthing their potential roles as biomarkers or therapeutic targets.

## Data Availability

Not applicable.

## Abbreviations

AI	Aromatase Inhibitor
AKT	Ak strain Transforming gene
ANRIL	Antisense Non-coding RNA in the INK4 Locus
ASOs	Antisense Oligonucleotides
BC	Breast Cancer
BrCSCs	Breast Cancer Stem-like Cells
c-MET	Mesenchymal Epithelial Transition cellular oncogene
CBP	cAMP-regulated-enhancer (CRE)-binding protein (CREB)-Binding Protein
CDK2	Cyclin-dependent Kinase 2
CDK4-6	Cyclin-dependent Kinase 4-6
ceRNA	Competing endogenous RNA
ChIP-Seq	Chromatin Immunoprecipitation Sequencing
ChIRP	Chromatin Isolation by RNA Purification
CLIP	Cross-Linking Immunoprecipitation
CRISPR/Cas9	Clustered Regularly Interspaced Short Palindromic Repeats-CRISPR associated protein 9
DES	Diethylstilbestrol
DO-RIP-seq	Digestion-Optimized RNA Immunoprecipitation cDNA library sequencing
DSCAM-AS1	Down Syndrome Cell Adhesion Molecule antisense1
DUSP7	Dual Specificity Phosphatase 7
E2F1	E2F transcription factor 1
EED	Embryonic Ectoderm Development
EFEMP1	EGF-containing fibulin-like extracellular matrix protein 1
EGR1	Early Growth Response 1
EMSA	Electrophoretic mobility shift assay
EMT	Epithelial-mesenchymal transition
EREs	Estrogen Response Element
ERINA	Estrogen inducible lncRNA
ERK	Extracellular signal-Regulated Kinase
eRNAs	Enhancer RNAs
ERα	Estrogen Receptor α
ERα+	Estrogen Receptor α positive
ESR1	Estrogen Receptor 1
ET	Endocrine Therapy
ETR	Endocrine Therapy Resistance
EZH2	Enhancer of Zeste Homolog 2
FOXO3	Forkhead Box O3
GRO-Seq	Global Run-On sequencing
GTI-seq	Global Translation Initiation sequencing
H3K27Ac	Histone 3 lysine 27 acetylation
H3K4	Histone H3 lysine K4
HB	Histidine-Biotin
HBD	Hormone Binding Domain
HDAC	Histone Deacetylase
HER2	Human Epidermal growth factor Receptor 2
HGF	Hepatocyte Growth Factor
hnRNPL	Heterogeneous nuclear Ribonucleoprotein L
HOTAIR	HOX Transcript Antisense RNA
ICI	Fulvestrant, ICI 182,780
KDM2A	Lysine demethylase 2A
linc-ROR	Long Intergenic Non-Protein Coding RNA, Regulator Of Reprogramming
LINC00472	Long Intergenic Non-Protein Coding RNA 472
LINC01016	Long Intergenic Non-Protein Coding RNA 1016
LMTK3	Lemur Tyrosine Kinase 3
LNA	Locked nucleic acid-modified oligonucleotides
lncRNA H19	Long non-coding RNA H19
lncRNA ROR	Long non-coding RNA Regulator Of Reprogramming
lncRNAs	Long non-coding RNAs
LPI	LncRNA-Protein iInteraction
LSD1	Lysine-Specific Demethylase 1
MAFG-AS1	MAF BZIP Transcription Factor G -AS1
MAPK	Mitogen-activated protein kinase
MEK	Mitogen-activated protein kinase kinase
MIAT	Myocardial Infarction-Associated Transcript
miR-155-5p	microRNA-155-5p
miR-339-5p	microRNA-339-5p
MIR2052HG	MIR2052 Host Gene
MLL1	Mixed Lineage Leukemiaprotein-1
MLL3	Mixed Lineage Leukemiaprotein-3
MLLs	Mixed Lineage Leukemias
mRNA	messenger RNA
mTOR	Mammalian Target Of Rapamycin
ncRNAs	Non-coding RNAs
NGS	Next-generation sequencing
NMR	Nuclear Magnetic Resonance
ORF	Open Reading Frame
PI3K	Phosphatidylinositol-3 Kinase
PKC	Protein Kinase C
PR	Progesterone Receptor
PRC2	Polycomb Repressive Complex 2
PTM	Post-Translational Modification
qPCR	Quantitative PCR
R3C	RNA chromosome conformation capture
RB1	Retinoblastoma protein 1
RNA Pol II	RNA Polymerase II
RNA-FISH	RNA-Fluorescent In Situ Hybridization
RNA-Seq	RNA Sequencing
RSK1	Ribosomal S6 Kinase 1
SAS	Small-angle Scattering
SERDs	Selective Estrogen Receptor Down-regulators
SERMs	Selective Estrogen Receptor Modulators
shRNAs	Short hairpin RNAs
siRNAs	Small interfering RNAs
sncRNAs	Small non-coding RNAs
STORM	Stochastic Optical Reconstruction mMicroscopy
SUZ12	Suppressor of Zeste 12 homolog
TDG	Thymine DNA glycosylase
TM4SF1	Transmembrane-4 L-six family member-1
TMPO	Thymopoietin
TMPO-AS1	TMPO antisense RNA1
TRIBE	Targets of RNA-binding proteins Identified By Editing
UCA1	Urothelial Carcinoma-Associated protein1
XIST	X-Inactive Specific Transcript
ZEB1	Zinc Finger E-Box Binding Homeobox 1
ZEB2	Zinc Finger E-Box Binding Homeobox 2
